# Turbo Gradient and Spin-Echo BLADE-DWI for Extraocular Muscles in Thyroid-Associated Ophthalmopathy

**DOI:** 10.3390/jcm12010344

**Published:** 2023-01-01

**Authors:** Qing Fu, Dingxi Liu, Hui Ma, Kun Zhou, Ting Yin, Chuansheng Zheng, Ziqiao Lei

**Affiliations:** 1 Department of Radiology, Union Hospital, Tongji Medical College, Huazhong University of Science and Technology, Wuhan 430022, China; 2 Hubei Province Key Laboratory of Molecular Imaging, Wuhan 430022, China; 3 Department of Digitalization, Siemens Shenzhen Magnetic Resonance Ltd., Shenzhen 518000, China; 4 MR Collaborations, Siemens Healthineers Ltd., Chengdu 610400, China

**Keywords:** diffusion-weighted imaging, apparent diffusion coefficient, extraocular muscles, thyroid-associated ophthalmopathy, turbo gradient and spin-echo BLADE-diffusion-weighted imaging (TGSE-BLADE-DWI), readout-segmented echo-planar imaging (RESOLVE)

## Abstract

**Purpose:** To investigate feasibility and diagnostic performance of turbo gradient and spin-echo BLADE (proprietary name for Periodically Rotated Overlapping ParallEL Lines with Enhanced Reconstruction [PROPELLER] in Siemens MR systems)-diffusion weighted imaging (TGSE-BLADE-DWI) for depicting extraocular muscle (EOM) involvement and activity in thyroid-associated ophthalmopathy (TAO), and to compare TGSE-BLADE-DWI with readout-segmented echo-planar imaging (RESOLVE). **Materials and methods:** Thirty-five patients with identified TAO underwent the two DWI scans. Two radiologists visually scored the image quality of the two DWIs with respect to the susceptibility artifacts and geometric distortions on a three-point scale. The maximum size (Size_max_) of EOMs and corresponding ADCs (cADCs) of each patient were compared between the active and inactive phases. The clinical activity score (CAS) was used as a reference to assess the diagnostic performance of EOM ADCs for grading TAO activity. ROC analysis, Pearson correlation, and Wilcoxon signed-rank test were used for statistical analyses. **Results:** For scores of EOMs, the image quality of TGSE-BLADE-DWI was significantly higher than that of RESOLVE. There were no statistically significant differences between the AUCs of the two DWIs, Size_max_, or cADCs between the active and inactive phases. TGSE-BLADE-DWI ADCs were significantly higher than the RESOLVE ADCs in the right superior rectus, right lateral rectus, left superior rectus, and left inferior rectus. There were no statistically significant correlations between the cADC or Size_max_, and CAS. The highest AUC was 0.697 for RESOLVE and 0.657 for TGSE-BLADE-DWI. The best performing ADC threshold was 1.85 × 10^−3^ mm^2^/s with 85.7% sensitivity, 58.8% specificity and 66.67% accuracy for RESOLVE and 1.99 × 10^−3^ mm^2^/s with 79.0% sensitivity, and 55.6% specificity and 65.27% accuracy for TGSE-BLADE-DWI. **Conclusion:** Compared to RESOLVE, TGSE-BLADE-DWI provided improved image quality with fewer susceptibility artifacts and geometric distortions for EOM visualization and showed an equivalent performance in detecting active TAO.

## 1. Introduction

Thyroid-associated ophthalmopathy (TAO), also known as Graves’ orbitopathy, is an autoimmune inflammatory disease of the orbit. Usually associated with thyroid disease, it is common among adults with hyperthyroidism, with about 20% of patients displaying different degrees of ocular signs and symptoms [1]. The most prominent symptom is the enlargement and swelling of the extraocular muscles (EOMs), of which the medial and inferior muscles are most frequently affected [2]. Even though the pathogenesis of TAO remains unclear, the natural history of this disease includes an active inflammatory phase and a chronic, inactive fibrotic phase. Accurate recognition of TAO staging is crucial for making proper clinical management decisions because early anti-inflammatory treatment or radiotherapy are useful in the active phase, and those with non-inflammatory TAO (inactive phase) may be treated by reconstructive and rehabilitative surgery [3].

Currently, the clinical activity score (CAS) [4,5] is universally used as a guideline for clinicians to define the active phase with higher scores, which reflects higher TAO activity, and this method is easy to implement in clinical practice. However, CAS scoring greatly depends on objective experience and only evaluates the superficial structures of the anterior orbital part. Moreover, a lower CAS score may not preclude the possibility of active TAO and does not exclude favorable therapeutic results from immunosuppressive treatment [5]. In a study by Mourits et al. [5], 36% of patients with CAS < 4 had a favorable response to immunosuppressive treatment, showing that CAS was not very sensitive. However, when the disease remained at the early active phase, CAS presented with a normal score with obvious inflammatory changes in the deep orbital structures, and thus led to misjudgment and delays for timely therapies. Each EOM could be involved in different phases of TAO (active or inactive stages). However, CAS alone may not have allowed for a precise assessment of the inflammatory or fibrotic phases of each EOM’s involvement. Therefore, the combination of other parameters for activity evaluation is recommended [5].

With the advantages of high-image resolution for soft tissues, magnetic resonance imaging (MRI) has been widely used in orbital imaging. Diffusion-weighted imaging (DWI) can noninvasively probe the microscopic movement of water molecules at the tissue and cellular level qualitatively and quantitatively and has been applied to assess EOM involvement in TAO [6,7,8,9]. An increased apparent diffusion coefficient (ADC) of inflamed EOMs was found in TAO patients compared to healthy controls [8,9], and the ADC changes of involved EOMs were observed prior to anatomical alternations on routine orbital MRI [9]. The increased ADC in the inflamed EOMs was associated with edema in the active phase of inflammation. DWI ADC could also be used to evaluate the treatment response for various diseases, including TAO [10,11].

TAO patients usually present with multiple and symmetric EOM enlargement in the bilateral orbits; the medial and inferior rectus muscles have been reported to be the most frequently affected EOMs. Moreover, asymmetric extraocular muscular involvement can also occur in clinical settings. In the early stages of TAO disease, the EOMs may not present with enlargement or edema. However, it is challenging to depict each EOM on DWI images due to the specific anatomical structures of the orbit. In addition, conventional DWI of EOMs using single-shot echo-planar (SS-EPI-DWI) is strongly affected by susceptibility artifacts and geometric distortions in regions with abundant air–bone–tissue interfaces and suffers from image blurring caused by signal decay in the long EPI readout window. Therefore, it is usually difficult to evaluate EOMs with the unsatisfactory image quality from SS-EPI-DWI [8].

Compared to SS-EPI-DWI, readout-segmented echo-planar imaging (RESOLVE) was proven to reduce susceptibility artifacts and geometric distortions [12,13], which were attributed to the effectively reduced echo spacing and echo time because the k-space has been divided into multiple segments along the readout direction in RESOLVE acquisition [14]. However, those artifacts could not be removed entirely due to their EPI intrinsic characteristic [12,13,15], particularly in regions with solid B_0_ inhomogeneities. In addition, the medial and inferior rectus muscles are more prone to be affected by susceptibility artifacts and geometric distortions, which can easily lead to an inaccurate ADC evaluation in ROI-based measurements on RESOLVE images [10]. Therefore, the clinical applications of non-EPI-based DWI methods have been raised for depicting TAO activity in some reports [11,16].

Turbo gradient and spin-echo BLADE (proprietary name for Periodically Rotated Overlapping ParallEL Lines with Enhanced Reconstruction [PROPELLER] in Siemens MR systems)-diffusion-weighted imaging (TGSE-BLADE-DWI), based on the TGSE sequence, acquires the k-space with a BLADE trajectory, and can provide images with significantly less geometric distortion and fewer susceptibility artifacts. The internal design of TGSE-BLADE-DWI was introduced by Li et al. [17], Zhou K et al. [18], and Hu et al. [19]. It has been used to assess middle ear cholesteatomas [15,20], optic neuritis [21], brain imaging in children [19], sinonasal lesions [22], orbital tumors [23], and cerebellopontine angle (CPA) lesions [24]. In addition, supportive data were significantly better than EPI-based DWI methods. However, to date, no studies have evaluated TGSE-BLADE-DWI for depicting the eight EOMs (right superior rectus, RSR; right inferior rectus, RIR; right medial rectus, RMR; right lateral rectus, RLR; left superior rectus, LSR; left inferior rectus, LIR; left medial rectus, LMR; and left lateral rectus, LLR) in TAO patients.

Therefore, the purpose of the current study was to investigate the feasibility of TGSE-BLADE-DWI for visualization of EOMs and evaluate its diagnostic performance in diagnosing TAO activity compared with RESOLVE in clinical settings.

## 2. Materials and Methods

### 2.1. Study Population

The Medical Ethics Committee of Tongji Medical College, Huazhong University of Science and Technology, approved this prospective study and written informed consent was obtained before the MRI examinations.

Thirty-five patients (a total of 70 unilateral eyes) with a clinical diagnosis of TAO (11 men, 24 women; mean age, 47.4 ± 10.2 years) were prospectively enrolled to undergo orbital RESOLVE and TGSE-BLADE-DWI between 1 September 2020 and 31 March 2022. The enrolled patients presented no other orbital pathologies. CAS scores were obtained by referring clinicians prior to orbital MRI scans for symptoms including spontaneous retrobulbar pain, pain on attempted up- or down-gaze, redness of the eyelids, redness of the conjunctiva, swelling of the eyelids, inflammation of the caruncle and/or plica, and conjunctival edema [25]. Patients with a CAS score ≥ 3/7 were identified as having active TAO [4,5]. Of the patients, seven did not receive treatment, four were treated with radiation therapy, nine had oral Selenium supplements (100–200 mcg twice daily), thirteen underwent immunosuppressive treatment with methylprednisolone (MTP) based on clinical indications, and six were treated with reconstructive and rehabilitative surgeries to relieve the corresponding clinical symptoms. The exclusion criteria were patients who refused to undergo the additional TGSE-BLADE-DWI, patients without clinically diagnosed TAO, and patients with non-MR compatible pacemakers, metallic implants, and an estimated glomerular filtration rate with less than 60 mL/min/1.73 m^2^.

### 2.2. Magnetic Resonance Imaging Protocol

All MRI examinations were performed on a 3T MR scanner (Magnetom Skyra, Siemens Healthcare, Erlangen, Germany) equipped with a 45 mT/m achievable gradient strength and 200 T/m/s maximum slew rate. A dedicated four-channel coil (Flex Small 4) was used to cover the bilateral orbits. Conventional MRI was required using the following sequences: coronal, axial, and oblique sagittal T2-weighted imaging (T2WI) with fat suppression. The detailed parameters were as follows: TR/TE: 5220/37 ms, FOV: 200 × 200 mm^2^, bandwidth: 220 Hz/pixel, slice thickness: 3 mm, flip angle: 160°, resolution 320 × 320, acquired voxel size = 0.63 × 0.63 × 3.0 mm^3^; axial and coronal T1-weighted imaging (T1WI) without fat suppression, TR/TE: 600/6.4 ms, FOV:180 × 180 mm^2^, bandwidth: 391 Hz/pixel, slice thickness: 3 mm, flip angle = 150°, and acquired voxel size = 0.35 × 0.35 × 3.0 mm^3^. If contrast-enhanced orbital MR imaging was demanded clinically, axial T1WI scanned by volume interpolated body examination (VIBE) with fat suppression before and after administration of contrast material would be scanned. The detailed parameters were as follows: TR/TE:18/3.69 ms, FOV:180 × 180 mm^2^, bandwidth: 180 Hz/pixel, slice thickness: 1 mm, flip angle = 9°, and acquired voxel size = 0.63 × 0.63 × 1 mm^3^.

RESOLVE and a prototype TGSE-BLADE-DWI were scanned with comparable spatial resolution and similar voxel sizes for all enrolled patients. The detailed TGSE-BLADE-DWI parameters were as follows: TR/TE: 4400/66 ms, FOV: 230 × 230 mm^2^, bandwidth: 650 Hz/pixel, echo spacing: 10.4 ms, voxel size: 1.4 × 1.4 × 2.5 mm^3^, number of slices: 16, matrix size: 160 × 160, BLADE coverage: 233.3%, phase encoding direction: R-L, diffusion encoding mode: 4-scan-trace, b-values: 0 and 600 s/mm^2^, averages: 1 for b = 0 s/mm^2^ and 2 for b = 600 s/mm^2^, turbo factor: 15, EPI factor: 3, and acquisition time: 4 min 45 s.

The detailed parameters for RESOLVE were as follows: TR/TE: 6000/61 ms, FOV: 200 × 200 mm^2^, bandwidth: 781 Hz/pixel, echo spacing: 0.4 ms, readout segment: 5, voxel size: 1.3 × 1.3 × 2.5 mm^3^, number of slices: 16, matrix size: 160 × 160, diffusion encoding mode: 4-scan-trace, b-values: 0 and 600 s/mm^2^, averages: 1 for b = 0 s/mm^2^, and 2 for b = 600 s/mm^2^, GRAPPA was used with acceleration factor 2, and acquisition time: 3 min 32 s. Corresponding ADC maps of the two DWIs were generated automatically after sequence scanning.

### 2.3. Subjective Evaluations

For each patient, EOMs of two DWIs were evaluated in the following order: RSR, RIR, RMR, RLR, LSR, LIR, LMR, and LLR. Overall, 280 orbital muscles were evaluated in 35 patients in the current study. Due to the proximity of the superior rectus and levator palpebrae on the images, these muscles were evaluated together where the superior rectus was used.

**Figure 1:**
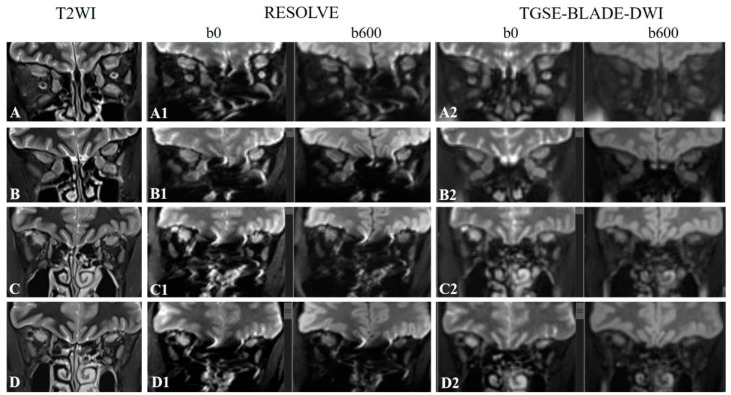
Representative coronal T2-weighted imaging (T2WI) images (**A**–**D**), coronal RESOLVE DWI b0 and b600 images (**A1**–**D1**), and coronal TGSE-BLADE-DWI b0 and b600 images (**A2**–**D2**) of a 54-year-old female patient with CAS = 4 (**A**, **A1**, **A2**, and **B**, **B1**, **B2**) and a 50-year-old male patient with CAS = 3 (**C**, **C1**, **C2** and **D**, **D1**, **D2**). For slice 1 (**A**, **A1** and **A2**), the subjective scores for the right superior rectus, right inferior rectus, right medial rectus, right lateral rectus, left superior rectus, left inferior rectus, left medial rectus, and left lateral rectus were 3, 2, 2, 2, 3, 2, 1, and 2 for RESOLVE and 3, 2, 3, 1, 3, 3, 2, and 1 for TGSE-BLADE-DWI, respectively. For slice 2 (**B**, **B1** and **B2**), the subjective scores for the EOMs were 3, 3, 2, 1, 3, 1, 1, and 1 for RESOLVE and 3, 3, 3, 1, 3, 3, 3, and 2 for TGSE-BLADE-DWI, respectively. For slice 3 (**C**, **C1** and **C2**), the subjective scores for the EOMs were 3, 1, 1, 3, 3, 1, 2, and 3 for RESOLVE and 3, 2, 2, 1, 3, 3, 3, and 3 for TGSE-BLADE-DWI, respectively. For slice 4 (**D**, **D1** and **D2**), the subjective scores for the EOMs were 3, 1, 1, 2, 3, 1, 1, and 3 for RESOLVE and 3, 1, 1, 1, 3, 3, 3, and 2 for TGSE-BLADE-DWI, respectively.

For the coronal slices from the region posterior to the globe and anterior to the point at which the EOMs were indistinguishable, two experienced radiologists (with sixteen and eight years of experience in neuroradiology, respectively) scored the image quality of two DWIs for depicting the 280 EOMs for susceptibility artifacts and geometric distortions using a three-point scale (3: excellent, EOM was clearly displayed with no susceptibility artifacts or geometric distortions; 2: moderate, EOM was displayed with slight susceptibility artifacts and geometric distortions; 1: poor, EOM was poorly displayed with severe susceptibility artifacts and geometric distortions) (Figure 1). A consensus score for each muscle was made when their findings were inconsistent, and a third reader (with 30 years of MRI experience) would then make the final decision. Both primary radiologists understood that the superior rectus represented a combination of the superior rectus and the levator palpebrae on each side because it is difficult to separate these two muscles in imaging. Therefore, only images with acceptable image quality scores (≥ 2) were used for the ADC measurements.

### 2.4. Objective Evaluations

Two pairs of region-of-interest (ROI) for each patient were delineated for quantitative assessment. First, among the 280 EOMs with acceptable DWI image quality scores (≥2), a circular ROI was placed on both ADC maps obtained from RESOLVE and TGSE-BLADE-DWI. The mean ADC values generated from the two DWIs for each EOM were recorded for comparison.

Second, for each patient, one ROI was delineated on the maximum cross-section in all eight EOMs on the coronal T2WI-short Tau inversion recovery (STIR) image, and the ROI area was recorded as Size_max._ Then, ROI was placed on the ADC maps of the cross-section of the same muscle, and the ROI was manually adjusted to avoid pixels with image distortions, artifacts, and rectus margins. The corresponding mean ADC values on RESOLVE and TGSE-BLADE-DWI images were recorded as cADC. Finally, the Size_max_ and cADC of active and inactive TAO were compared, and the correlations between cADC and Size_max_, and cADC and CAS grading were also evaluated.

### 2.5. Statistical Analysis

Continuous variables were presented as the means ± standard deviation (SD). If the ADCs of the 280 EOMs between RESOLVE and TGSE-BLADE-DWI, Size_max_, and cADCs between the active and inactive phases conformed to a normal distribution, the paired t-test was used. Otherwise, the Wilcoxon signed-rank test was used. Correlations of cADC and Size_max_ and CAS were evaluated by the Pearson correlation. Receiver operating characteristics (ROC) curves were used to evaluate the diagnostic performance of the ADC values of each EOM for differentiating active and inactive TAO, and the method presented by Delong et al. [26] was used to compare the area under curves (AUCs) of the two DWIs with 95% confidence intervals (CIs). Statistical analyses were performed with SPSS software (IBM SPSS 22, IBM Corp., Armonk, NY, USA) and MedCalc software (version 18.11.3; MedCalc Software, Ltd.). A *p*-value < 0.05 was considered to be statistically significant.

## 3. Results

Of the 35 enrolled patients, 21 underwent orbital MR scanning without injected contrast, and 14 were scanned with contrast-enhanced T1WI, according to the clinical requests.

### 3.1. Subjective Evaluations

**Figure 2:**
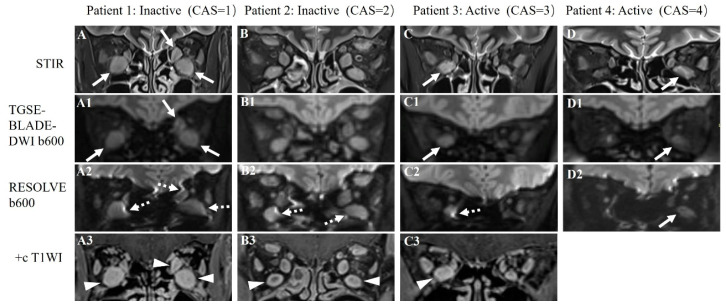
Representative STIR (**A**–**D**), TGSE-BLADE-DWI b600 (**A1**–**D1**), and RESOLVE b600 images (**A2**–**D2**) of four TAO patients with different clinical activity scores (CAS). Patient 1 was a 43-year-old male TAO patient with CAS = 1, with bilateral enlarged inferior and left medial rectus muscles shown on the STIR sequence (**A**) that could be clearly displayed on TGSE-BLADE-DWI (white arrows in **A1**); slight to moderate susceptibility artifacts and geometric distortions were seen with RESOLVE (dotted arrows in **A2**). Patient 2 was a 45-year-old male TAO patient with CAS = 2; the bilateral superior, inferior, and medial rectus muscles were enlarged in size and were clearly visualized without artifacts on TGSE-BLADE-DWI (**B1**); the bilateral inferior rectus muscles were slightly distorted on RESOLVE (dotted arrows in **B2**). Patient 3 was a 42-year-old female TAO patient with CAS = 3; the right inferior rectus was enlarged on STIR (**C**) and is clearly shown on TGSE-BLADE-DWI (white arrow in **C1**) but was severely affected by susceptibility artifacts and geometric distortions on RESOLVE (dotted arrow in **C2**). Patient 4 was a 55-year-old female TAO patient with CAS = 4; the left inferior rectus was enlarged on STIR and was displayed well both in TGSE-BLADE-DWI and RESOLVE (white arrows in **D1**, **D2**). Contrast-enhanced T1WI (+c T1WI) scans (**A3**–**C3**) were performed in patient 1–3, and the coronal images identified the enlargement of multiple EOMs (arrowheads in **A3**, **B3** and **C3**).

Of all the EOMs (*n* = 280), 73.6% (206/280) produced an acceptable image quality, with scores ≥ 2 on RESOLVE imaging, whereas 92.5% (259/280) of EOMs were scored to be acceptable on TGSE-BLADE-DWI imaging. As shown in Table 1, significantly higher objective scores were obtained from TGSE-BLADE-DWI images with less geometric distortions and susceptibility artifacts compared to RESOLVE images for depicting all the EOMs, with the exception being the right lateral rectus (Figure 2).

### 3.2. Objective Evaluations

Overall, when the left and right EOM data from each patient were combined as the superior rectus, inferior rectus, lateral rectus, and medial rectus muscles, the analyses showed that there were no statistically significant differences in the ADCs between RESOLVE and TGSE-BLADE-DWI in the superior rectus (z = −5.965, *p* < 0.001), inferior rectus (z = −2.949, *p* = 0.003), and lateral rectus (z = −3.105, *p* = 0.002), with the exception being the medial rectus (z = −0.660, *p* = 0.509).

Furthermore, the mean ADCs of TGSE-BLADE-DWI were significantly higher than the values calculated on the RESOLVE images in the right superior rectus (*p* < 0.001), right lateral rectus (*p* = 0.006), left superior rectus (*p* = 0.006), and left inferior rectus (*p* = 0.004), and showed with no differences in other EOMs between the two DWIs (Table 2).

**Figure 3:**
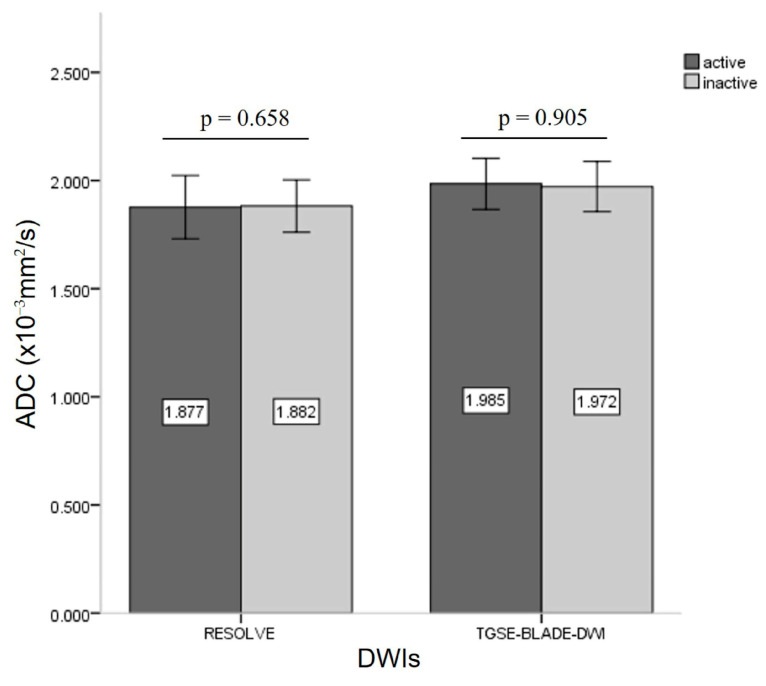
Bar graph demonstrating the comparison of ADC values between active and inactive TAO in the two DWIs.

**Figure 4:**
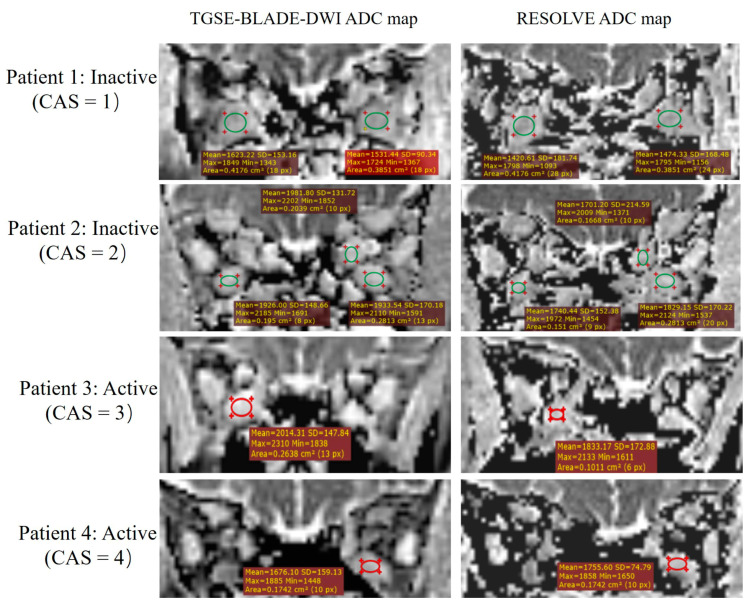
The detailed ADC measurements of the same four patients listed in Figure 2. The placement of ROIs in the two DWIs should be noted to avoid artifacts and the EOM margins.

According to the CAS grading, 12 patients were grouped into active TAO with CAS ≥ 3 (three men, nine women; mean age, 47.8 ± 7.5 years), and 23 patients (eight men, fifteen women; mean age, 46.7 ± 11.4 years) were identified as inactive TAO with CAS < 3. To differentiate active and inactive TAO, there were no significant differences in Size_max_ (74.3 ± 9.32 mm^2^ vs. 82.6 ± 23.56 mm^2^; z = −0.674, *p* = 0.500), cADCs in RESOLVE (z = −0.443, *p* = 0.658), or cADCs in TGSE-BLADE-DWI (z = −0.119, *p* = 0.905) (Figure 3). There were no statistically significant correlations between cADC and Size_max_ (RESOLVE: r = 0.035, *p* = 0.866; TGSE-BLADE-DWI: r = 0.166, *p* = 0.419), and CAS (RESOLVE: r = −0.035, *p* = 0.796; TGSE-BLADE-DWI: r = −0.015, *p* = 0.914). The mean cADCs obtained from TGSE-BLADE-DWI were significantly higher than those of RESOLVE for both the active phase (1.98 ± 0.311 vs. 1.88 ± 0.38; z = −2.692, *p* = 0.007) and inactive phase (1.97 ± 0.30 vs. 1.88 ± 0.32; z = −2.195, *p* = 0.028) (Figure 4).

There was no statistical difference in the AUCs for RESOLVE and TGSE-BLADE-DWI (z = 0.054, *p* = 0.957). Of the eight EOMs, the best AUC was 0.697 for RESOLVE in the right medial rectus and 0.657 for TGSE-BLADE-DWI in the left superior rectus. The best performing ADC threshold was 1.85 × 10^−3^ mm^2^/s, with 85.7% sensitivity, 58.8% specificity, and 66.67% accuracy for RESOLVE; however, it was 1.99 × 10^−3^ mm^2^/s, with 79.0% sensitivity, 55.6% specificity and 65.27% accuracy for TGSE-BLADE-DWI.

## 4. Discussion

This study demonstrated the feasibility of TGSE-BLADE-DWI for depicting EOMs in TAO patients and its diagnostic performance for distinguishing active and inactive TAO in clinical use. The data suggest that TGSE-BLADE-DWI is superior to RESOLVE in improved image quality with decreased susceptibility artifacts and fewer geometric distortions. In addition, TGSE-BLADE-DWI showed comparable performance to RESOLVE in detecting active TAO. However, with the limited AUCs obtained from both TGSE-BLADE-DWI and RESOLVE for distinguishing active and inactive TAO, further investigations are needed for validation.

In orbital DWI, TGSE-BLADE-DWI showed fewer susceptibility artifacts and geometric distortions than RESOLVE, which is crucial for accurately depicting EOMs, particularly for those located in the vicinity of air–bone–soft tissue interfaces, such as the medial and inferior rectus muscles. As reported in previous studies [2,6], the most frequently affected EOM was the medial rectus muscle, followed by the inferior rectus muscle. Accurately displaying these anatomical structures is difficult using conventional EPI-based DWI methods as the region is severely affected by susceptibility artifacts and geometric distortions, even with the advanced RESOLVE method. To decrease the interference of these artifacts in the display of anatomical structures and lesion conspicuity, several researchers have investigated the clinical usefulness of non-EPI-based DWI methods, such as TGSE-BLADE-DWI. Sheng et al. [15] and Li et al. [20] applied both RESOLVE and TGSE-BLADE-DWI in diagnosing cholesteatoma. Their results revealed that TGSE-BLADE-DWI outperformed RESOLVE in detecting cholesteatomas, particularly for small lesions < 2–3 mm. Evaluation of cholesteatomas of this size benefits from improved image quality by reducing B_0_ inhomogeneity-related artifacts and blurring TGSE-BLADE-DWI images.

Two recent papers [22,23] focused on comparing RESOLVE and TGSE-BLADE-DWI use on orbital tumors and sinonasal lesions, with minimal subjective scores and quantitative evaluations for geometric distortions through the geometric distortion rate (GDR) parameter, supporting the superiority of TGSE-BLADE-DWI for depicting these lesions. Moreover, TGSE-BLADE-DWI has been used to visualize anatomic cerebellopontine angle (CPA) structures and identify CPA tumors, by comparing RESOLVE and SS-EPI-DWI, as in the study by Fu et al. [24]. The researchers concluded that TGSE-BLADE-DWI demonstrated improved image quality with minimal geometric distortions and ghosting artifacts to depict CPA tumors with better lesion conspicuity. Consistent with other published research, we found that TGSE-BLADE-DWI exhibited better image quality than RESOLVE in diffusion-weighted imaging for displaying anatomical EOM structures in TAO patients with a distortion-free advantage, particularly for the medial and inferior rectus muscles. This finding is important for the accurate quantitative ADC measurement of the involved muscles.

In DWI, the set of b-values varies in different studies for TAO evaluation. In Politi et al.’s report [8], b-values of 0 and 700 s/mm^2^ were used for TAO in SS-EPI-DWI. The values of 0 and 1000 s/mm^2^ were used in some studies [9,11], and 0, 500, and 1000 s/mm^2^ were applied in a 2017 paper [6], whereas 0 and 500 s/mm^2^ were applied in a 2018 paper [7]. In the current study, we applied 0 and 600 s/mm^2^ in two DWIs to obtain a higher signal-to-noise ratio (SNR) of EOMs in the coronal orientation and to compensate for the intrinsic lower SNR efficiency of the TGSE-BLADE-DWI sequence due to the gradient component within the sequence design [19]. This finding would be helpful for the clear display of EOMs and credible subjective evaluation of TGSE-BLADE-DWI in the current study.

During the acute inflammatory and edematous phases of TAO, the muscle usually presents with swelling and enlargement. However, in the current study, no significant differences in Size_max_ were detected between the active and inactive phases. We assume that this was because enlarged muscles could present both in the active and inactive phases of TAO disease, and this is consistent with the findings of Hiwatashi et al. [7]. As illustrated in Figure 1 in the current study, multiple enlarged EOMs were seen in patients with different clinical activity scores, including the active phase (patients 1 and 2) and inactive phase (patients 3 and 4).

Conventionally, clinical CAS is regarded as the ‘gold standard’ in clinical practice, though it shows limited accuracy. Therefore, clinical CAS was still used in this study as the reference standard to determine TAO activity. Theoretically, ADC measurements of EOMs during active inflammation should be increased, opposite to those in fibrotic EOMs. This finding reflects the different pathological changes of the two phases; in addition, the ADCs of TAO patients have been reported to be significantly higher than those of healthy controls [8,9]. However, Politi et al. [8] and Hiwatashi et al. [7] showed that DWI ADCs in the active and inactive phases had no statistically significant differences, and Kilicarslan et al. [9] further reported that there were no significant differences in the ADCs between uninvolved and involved muscles in the medial and lateral rectus muscles. Consistent with these published studies, our data also showed no significant differences in ADCs between the active and inactive TAO patients, either in RESOLVE or TGSE-BLADE-DWI.

Using the clinical CAS as a reference, TGSE-BLADE-DWI showed equivalent diagnostic performance in detecting active and inactive TAO compared to RESOLVE. ROC analyses in the current study revealed that even though the best AUC value of RESOLVE (0.697) in the right medial rectus was slightly higher than that of TGSE-BLADE-DWI (0.657) in the left superior rectus, there was no statistical difference in AUCs for those two DWIs, with 85.7% sensitivity and 58.8% specificity for RESOLVE, and 79.0% sensitivity and 55.6% specificity for TGSE-BLADE-DWI. Interestingly, as the most frequently affected EOM, both the left and right medial rectus muscle ADCs between the two DWIs showed no statistical difference, with *p*-values > 0.100. In contrast, the ADC comparison between the two DWIs showed different results in the eight EOMs. We considered the reason for these findings to be related to the following factors: (**a**) B_0_ and B_1_ inhomogeneities; (**b**) ADC of EOMs may vary according to different stages of their pathological nature and cellularity of tissues [27], which could be affected by various factors, such as edema, fibrosis, and the infiltration of inflammatory cells and deposition of glycosaminoglycan [8]; (**c**), the ROIs placed in EOMs were not the same size or in the exact location in TGSE-BLADE-DWI and RESOLVE. Since the EOM depiction in RESOLVE suffered from susceptibility artifacts and geometric distortions, the actual ROI in RESOLVE was adjusted to avoid those artifacts and was placed within the center of each muscle (Figure 3). Therefore, this may also cause differences in the ADC values between RESOLVE and TGSE-BLADE-DWI.

In the current study, the mean active and inactive ADCs in TGSE-BLADE-DWI (1.98 and 1.97 × 10^−3^ mm^2^/s) and RESOLVE (1.88 and 1.88 × 10^−3^ mm^2^/s) were similar to the data published by Politi et al. [8] (1.98 and 1.93 × 10^−3^ mm^2^/s). However, the diagnostic capacity of TGSE-BLADE-DWI and RESOLVE requires further validation. Therefore, we applied the active or inactive phases defined by CAS to all eight EOMs for each patient, resulting in some biases in the ROC analyses for both RESOLVE and TGSE-BLADE-DWI. This may have led to limited AUC values for the two DWIs (<0.700). However, we still concluded that the performance of TGSE-BLADE-DWI ADC and RESOLVE ADC is equivalent under the same level of this bias due to the absence of a standard reference for each muscle in the clinical setting. Moreover, we did not apply this strategy using the most inflamed muscle, proposed by Mayer et al. [28] because we believed it difficult to recognize the most inflamed muscle in TAO patients, particularly those with multiple enlarged EOMs. Furthermore, DWI ADC values maybe be helpful for monitoring treatment responses before and after radiation therapy [11,29]; therefore, we assumed that the long-term follow-up comparisons of ADCs in TGSE-BLADE-DWI before and after clinical treatment may be valuable to confirm the initial judgement for active or inactive phase assessment.

Our study had several limitations. First, sample size of enrolled patients was relatively small (*n* = 35); future studies with larger populations are needed. Second, we used the EOM with maximal cross-sectional area in the STIR-sequence as the representative muscle for measuring the corresponding ADC and Size_max_. This might have led to some bias because this area measured from STIR might have been greater than that obtained on T1 images, which might be related to the inflammatory water content within the EOM or orbital fat in the active phase and thus led to a more substantial anatomical size [28]. Finally, the clinical value of TGSE-BLADE-DWI needs further validation. The enlarged EOMs with T2 prolongation in TAO patients were reported to be representing muscle edema [30,31]. However, in the current study, we only assessed the DWI method for differentiating the active from inactive phases. The diagnostic accuracy of TGSE-BLADE-DWI combined with the T2 mapping technique for active assessment would need to be performed in future investigations. Advanced multi-parameter DWI models, such as diffusion tensor imaging (DTI) [32], might be helpful in the future. Moreover, intravoxel incoherent motion (IVIM) MRI has been reported to be useful to distinguish IgG4-related orbital disease from other causes of orbital inflammation [33]; therefore, IVIM may be a potential tool for depicting EOMs involvement. This will be explored in our next plan in order to increase the diagnostic accuracy for TAO activity definition.

In conclusion, TGSE-BLADE-DWI improved image quality with fewer susceptibility artifacts and geometric distortions for the visualization of EOMs than RESOLVE and showed equivalent performance in detecting active TAO. This modality might be an alternative to RESOLVE in TAO evaluation. However, further validation with a larger population and long-term follow-up for treatment responses are needed to identify the potential value of TGSE-BLADE-DWI in clinical settings.

## Figures and Tables

**Table 1:** Subjective image scores of EOMs for the 35 enrolled TAO patients [M(*Q*_1_, *Q*_3_)].

EOMs	RESOLVE	TGSE-BLADE-DWI	Z	*p*
Right superior rectus	2(1,3)	2(2,3)	−3.177	0.001
Right inferior rectus	1(1,2)	3(2,3)	−8.315	<0.001
Right medial rectus	1(1,2)	3(2,3)	−7.198	<0.001
Right lateral rectus	2(2,3)	2(1,3)	−0.089	0.929
Left superior rectus	2(1,3)	3(2,3)	−4.977	<0.001
Left inferior rectus	1(1,2)	3(3,3)	−8.458	<0.001
Left medial rectus	1(1,2)	3(2,3)	−8.692	<0.001
Left lateral rectus	2(1,3)	2(2,3)	−3.103	<0.001

EOMs, extraocular muscles; TAO, thyroid-associated ophthalmopathy; RESOLVE, readout-segmented echo-planar imaging; TGSE-BLADE-DWI, turbo gradient and spin-echo BLADE diffusion-weighted imaging; M(*Q*1, *Q*3) represented the median, first, and third quartiles.

**Table 2:** Comparison of ADC values of RESOLVE and TGSE-BLADE-DWI in EOMs (×10^−3^ mm^2^/s).

EOMs	RESOLVE	TGSE-BLADE-DWI	Z	*p*
Right superior rectus	1.81 ± 0.27 (1.37–2.52)	2.09 ± 0.24 (1.68–2.58)	−5.083	<0.001
Right inferior rectus	1.73 ± 0.30 (0.99–2.66)	1.76 ± 0.27 (1.08–2.56)	−1.127	0.260
Right medial rectus	1.92 ± 0.36 (1.31–2.92)	1.99 ± 0.28 (1.63–3.03)	−1.400	0.161
Right lateral rectus	1.98 ± 0.27 (1.39–2.55)	2.07 ± 0.28 (1.51–2.67)	−2.760	0.006
Left superior rectus	1.92 ± 0.32 (1.47–2.61)	2.02 ± 0.34 (1.46–2.92)	−2.737	0.006
Left inferior rectus	1.71 ± 0.19 (1.32–2.18)	1.79 ± 0.20 (1.31–2.33)	−2.865	0.004
Left medial rectus	1.87 ± 0.28 (1.18–2.39)	1.87 ± 0.20 (1.43–2.37)	−0.311	0.756
Left lateral rectus	2.04 ± 0.35 (1.33–2.89)	2.09 ± 0.30 (1.55–2.88)	−1.686	0.092

ADC, apparent diffusion coefficient; TAO, thyroid-associated ophthalmopathy; RESOLVE, readout-segmented echo-planar imaging; TGSE-BLADE-DWI, turbo gradient and spin-echo BLADE diffusion-weighted imaging; EOMs, extraocular muscles.

## Data Availability

The datasets generated during and/or analyzed during the current study are available from the corresponding author on reasonable request.
